# Das Handwerk zwischen Corona-Krise und Rückvermeisterung

**DOI:** 10.1007/s10273-021-2873-x

**Published:** 2021-06-23

**Authors:** Katarzyna Haverkamp, Petrik Runst, Till Proeger

**Affiliations:** Volkswirt. Inst. f. Mittelstand u. Handwerk, Universität Göttingen, Heinrich-Düker-Weg 6, 37073 Göttingen, Deutschland

**Keywords:** L26, L51, M13

## Abstract

Das Handwerk zeigte vor der Corona-Krise eine sehr positive Geschäftslage. Im Verlauf der Krise kam es zur Überlagerung der Effekte der Lockdown-Maßnahmen und der Novellierung der Handwerksordnung, durch die zwölf Handwerke erneut meisterpflichtig wurden. Mit Hilfe der Daten der Handwerkskammern werden die Effekte beider Veränderungen auf die Betriebsdynamik analysiert. Die Ergebnisse zeigen, dass das Handwerk in der Krise bisher keinen strukturellen Schaden — d. h. einen Rückgang an Gründungen und einen Anstieg an Betriebsaufgaben — aufweist. Gleichzeitig wirkt sich die Re-Regulierung auf die Gründungsdynamik in den betroffenen Handwerken aus.

## Abstract (en)

In the spring of 2020, the skilled crafts sector was simultaneously affected by two different policy measures: the COVID-19-induced lockdown measures and a new market entry regulation. We use administrative data from the German Chambers of Skilled Crafts to analyse the effects of both of these policy changes on firms’ registration rates. The results provide no evidence of possible structural damage in business formation as a consequence of corona measures. Instead, we find a reduction in entrepreneurial activity due to the new regulation policy consistent with prior expectations.

Zu den wesentlichen Merkmalen von Wirtschaftskrisen gehört, dass sie sich auf einzelne Wirtschaftsbereiche unterschiedlich stark auswirken (Heilemann, 2019; Bofinger et al., 2020; Thomä, 2011). Das Handwerk meldete vor der Corona-Krise eine ausgesprochen positive Geschäftslage (ZDH, 2019). Im ersten Quartal 2020 konnten die Umsätze weiter gesteigert werden: Die Handwerksberichterstattung zeigte ein Umsatzplus von 3,4 %. Im zweiten Quartal 2020 kam es zu einem tiefen Einbruch: Zum ersten Mal seit sieben Jahren verzeichnete das Handwerk einen Absatzrückgang. Die Umsätze gingen in den zulassungspflichtigen Handwerken um 7,4 %, in den zulassungsfreien Handwerken um 9,4 % zurück. Besonders tiefe Spuren hat die Krise im Kraftfahrzeuggewerbe (-21,6 %), im Gesundheitsgewerbe (-19,9 %) sowie bei den Konditor:innen (-29 %) und dem Friseurhandwerk (-24,8 %) hinterlassen. Die zulassungspflichtigen Bauhaupt- und Ausbaugewerbe konnten hingegen auch in der Corona-Krise die Umsätze gegenüber der Vorjahresperiode leicht steigern (+4,7 % und +1,1 %) (Statistisches Bundesamt, 2020a, 2020b, 2020c). Während in vielen Handwerksbranchen betriebliche Strategien zur Bewältigung der Umsatzrückgänge und der Liquiditätsengpässe entwickelt werden mussten, blieb die Beschäftigung weitgehend stabil: Die Beschäftigtenzahlen im zulassungspflichtigen Handwerk lagen im zweiten Quartal 2020 nur um 1,7 % unter dem Vorjahresquartal. Wie in der Wirtschaftskrise 2008/2009 reagierten die Betriebe auf die Umsatzrückgänge nicht mit einer Entlassungswelle, sondern hielten an ihren Beschäftigten fest. Erneut zeigte sich das beharrende Beschäftigungsverhalten des Handwerks bei Konjunktureinbrüchen (Thomä, 2011).

Die Auswirkungen der Corona-Krise auf Umsätze und Beschäftigte lassen sich mit den Quartalsdaten des Statistischen Bundesamts valilde beschreiben; es fehlen hingegen Informationen zur Betriebsdynamik. Amtliche Daten hierzu werden mit der Handwerkszählung für das laufende Jahr erst 2022 veröffentlicht (Statistisches Bundesamt, 2019). Eine kurzfristig verfügbare Datengrundlage zur Beschreibung des Gründungsgeschehens bietet die organisationseigene Statistik des Handwerks. Die Handwerkskammerverzeichnisse enthalten die tagesgenauen Angaben zur Gewerbean- und -abmeldung. Zentral ist die Frage, inwieweit die gesundheitspolitischen Maßnahmen zu verminderter Gründungstätigkeit und ausbleibenden Marktaustritten – und damit zu nachlassender Erneuerungs- und Innovationsfähigkeit des Handwerks – beigetragen haben. Zudem werden die ersten Effekte der Novellierung der Handwerksordnung im Februar 2020 betrachtet.

**Dr. Katarzyna Haverkamp**, **Dr. Petrik Runst** und **Dr. Till Proeger** sind wissenschaftliche Mitarbeiter:innen am Volkswirtschaftlichen Institut für Mittelstand und Handwerk an der Universität Göttingen.

## Betriebsdynamik im Konjunkturverlauf

Die Entrepreneurship-Forschung zeigt, dass sich die Gründungstätigkeit und Konjunktur oft reziprok verhalten: Bei Hochkonjunktur sinken die Gründungszahlen, während sie in den ökonomischen Krisen steigen. Diese inverse Beziehung hängt mit der Entwicklung des Arbeitsmarkts zusammen: Die Zunahme der Arbeitslosigkeit in Krisen trägt zur Erhöhung der Gründungszahlen bei, da Erwerbslose eine höhere Neigung zur Betriebsgründung aufweisen (Konon, Fritsch und Kritikos, 2018; Fossen 2020). Somit konnte zu Beginn der Corona-Krise erwartet werden, dass die konjunkturelle Eintrübung mit einer stärkeren Gründungsdynamik im Handwerk einhergeht – mit einer Verschiebung von Chancen- zu Notgründenden (Haverkamp, Proeger und Runst, 2020). Rasch zeigte sich jedoch, dass die Unternehmen auf die Krise nicht mit einer Entlassungswelle, sondern mit der intensiven Inanspruchnahme des Kurzarbeitsgelds reagierten, sodass der gesamtwirtschaftliche Anstieg der Arbeitslosigkeit und damit der Notgründungen vergleichsweise moderat blieben (Anger et al., 2020).

Entscheidend für die Entwicklung der Gründungsdynamik im Handwerk 2020 sollten daher zwei andere Faktoren werden. Zum einen war der Beginn der Krise mit einer starken Verunsicherung verbunden; die Beurteilung der Geschäftslage verschlechterte sich in einigen Handwerksbranchen massiv. So schätzten im zweiten Quartal 2020 nur noch 12 % der Betriebe aus dem Gesundheitsgewerbe die Geschäftslage als gut ein, im Kraftfahrzeuggewerbe 17 % und im Lebensmittelhandwerk 25 %. Nur im Bau- und Ausbaugewerbe berichteten mehr Unternehmen von einer positiven als von einer negativen Geschäftslage (ZDH, 2020). Da die Beurteilung der Geschäftslage die Gründungsabsichten beeinflusst (Giotopoulos, Kontolaimou und Tsakanikas, 2017), ist anzunehmen, dass die gestiegene Unsicherheit die Gründungszahlen reduzierte.

Zum anderen trat im Frühjahr 2020 die Novelle der Handwerksordnung in Kraft. Am 12. Dezember 2019 verabschiedete der Bundestag das Vierte Gesetz zur Änderung der Handwerksordnung. Hiermit wurden zwölf Handwerke aus dem Bereich der zulassungsfreien in den Bereich der zulassungspflichtigen Handwerke überführt.[1] Aus zahlreichen empirischen Untersuchungen zu den Effekten der letzten Novelle der Handwerksordnung von 2004 ist bekannt, dass die regulatorischen Veränderungen tiefe Spuren in den Gründungsstatistiken hinterlassen haben (Fredriksen et al., 2020). Damit war zu erwarten, dass die Wiedereinführung der Meisterpflicht zur Reduktion der Gründungszahlen im Handwerk führen würde. Das Änderungsgesetz trat zum 14. Februar 2020 in Kraft, sodass sich dessen Effekte mit den Effekten der Corona-Maßnahmen überlagern. Um diese Effekte zu trennen, werden für die Gruppe der rückvermeisterten Handwerke eine zusätzliche statistische Kategorie (Gruppe C) und für alle neu entstandenen Handwerksgruppen mit einem jeweils unterschiedlichen Zulassungsstatus neue konsistente Zeitreihen ab 2007 gebildet.

## Verwaltungsdaten der Handwerkskammern

Tagesgenaue Daten zur Betriebsdynamik im Handwerk können auf Basis der Unternehmensverzeichnisse der Handwerkskammern gewonnen werden. Nach § 6 und § 19 der Handwerksordnung (HwO) haben die Handwerkskammern Verzeichnisse zu führen, in die alle Handwerksbetriebe ihres Kammerbezirks eingetragen werden müssen. Zu jedem Betrieb liegen damit auf regionaler Ebene Daten zur An- und Abmeldung des Gewerbes, zur Eintragungsgrundlage, zum ausgeübten Handwerk sowie personenbezogene Angaben vor (Müller, 2010). Diese Daten werden auf der Bundesebene vom Zentralverband des Deutschen Handwerks (ZDH) aggregiert und als Halbjahresergebnisse bzw. Jahresergebnisse zusammengefasst veröffentlicht.[2] Der bei den Handwerkskammern vorliegende Datenbankbestand ermöglicht aber nicht nur eine halbjährliche, sondern auch eine unterjährige Erfassung aller Zu- und Abgänge. Für die Zwecke dieser Analyse wurden daher die betriebsbezogenen Daten von insgesamt 13 Kammern abgefragt und zusammengeführt. Erfasst wurden dabei alle Betriebe, die ab dem 1.1.2007 in die Verzeichnisse eingetragen bzw. aus diesen gelöscht wurden. Damit entstand ein harmonisierter Datensatz mit insgesamt 599.612 Beobachtungen.

## Gründungsdynamik und Erholungseffekte

Die Betrachtung der Eintragungszahlen im gesamten Zeitraum zwischen Januar 2007 und August 2020 ermöglicht es, einen längerfristigen Verlauf der Eintragungsdynamik nachzuzeichnen und damit die Veränderungsraten zwischen 2020 und 2019 besser einzuordnen. Abbildung 1 verdeutlicht, dass sich in der Meldestatistik der Handwerkskammern insgesamt ein wiederkehrender Jahresverlauf zeigt: Die meisten Anmeldungen werden zu Jahresbeginn vorgenommen, ab Februar bewegt sich die Zahl der monatlichen Meldungen auf dem Durchschnittsniveau des jeweiligen Jahres und sinkt anschließend im Dezember stark. Dieser Verlauf zeigt sich grundsätzlich auch 2020. Jedoch werden bei der Betrachtung von 2020 die tie-

1 Die Änderung betrifft die Fliesen-, Platten- und Mosaikleger, Betonstein- und Terazzohersteller, Estrichleger, Behälter- und Apparatebauer, Parkettleger, Rollladen- und Sonnenschutztechniker, Drechsler (Elfenbeinschnitzer) und Holzspielzeugmacher, Böttcher, Glasveredler, Schilder- und Lichtreklamehersteller, Raumausstatter sowie Orgel- und Harmoniumbauer. Das Holz- und Bautenschutzgewerbe und das Bestattungsgewerbe wurden zudem nicht mehr dem handwerksähnlichen Gewerbe (Anlage B2), sondern dem zulassungsfreien Handwerk (Anlage B1) zugeordnet (ausführlich Sallaberger, 2020).

2 www.zdh-statistik.de

Abbildung 1

**Eintragungen nach Monat und Jahr, Januar 2007 bis August 2020**

Quelle: Sonderabfrage bei 13 Handwerkskammern, August/September 2020.

fen Einschnitte ab März deutlich. Die Eintragungen liegen im März, April und Mai unter dem Niveau der Vorjahre. So betragen die Rückgänge der Gründungszahlen[3] im Vergleich zum Vorjahresmonat zwischen 2020 und 2019 -12 % (März), -17 % (April), -23 % (Mai). Ab Juni zeigen sich aber erste Erholungseffekte.

Auffällig sind ferner die im Vergleich unerwartet hohen Eintragungswerte ab September 2019. Diese gestiegene Gründungstätigkeit im Herbst und Winter 2019 lässt sich als Vorzieheffekt der Handwerksnovelle 2020 interpretieren. Die Novelle trat zwar zum 14. Februar 2020 in Kraft, wurde jedoch bereits im Vorjahr intensiv bei Anhörungen von Fachverbänden diskutiert (Bizer, Haverkamp und Proeger, 2019). Anzunehmen ist, dass die Konkretisierung der im Koalitionsvertrag festgehaltenen Absichten der Bundesregierung zur Wiedereinführung der Meisterpflicht in den betroffenen Handwerken die Neigung zur Gründung ohne Meisterbrief im Vorfeld der Novelle erhöht hat. So verzeichneten die hier betrachteten Kammern im Dezember 2019 41 % mehr Eintragungen als im Dezember des Vorjahrs.

## Rückvermeisterte Handwerke stark betroffen

Betrachtet man die Zeitreihen für 2019 und 2020 genauer und nimmt die Unterscheidung zwischen den Gruppen der unverändert zulassungspflichtigen Handwerke (Gruppe A), der zulassungsfreien Handwerke (Gruppe B1), der handwerksähnlichen Gewerbe (Gruppe B2) und der rückvermeisterten Handwerke (Gruppe C), dann lässt sich der Effekt der Wiedereinführung der Meisterpflicht isolieren. Die Zeitreihen sind dabei in Abbildung 2 als Indexzahlen dargestellt (1. Januarwoche 2020 = 100), um den Vergleich zwischen den Jahren und den Gruppen zu vereinfachen. Diese Art der Betrachtung verdeutlicht die unterschiedliche Entwicklung der Eintragungszahlen nach dem Zulassungsstatus. In der Gruppe der rückvermeisterten Handwerke (Gruppe C) lässt sich ein sehr deutlicher Rückgang der Eintragungsdynamik bereits ab Mitte Februar feststellen. Die absolute Zahl der Eintragungen in den zwölf rückvermeisterten Handwerken ist zwischen März und August von 3.241 (2019) auf 977 (2020), d. h. um 70 % gesunken. Die theoretisch erwarteten negativen Auswirkungen der höheren Zugangshürden auf die Gründungszahlen traten damit auf, wie erwartet.

Der Rückgang der Gründungsdynamik ab März 2020 lässt sich aber nicht allein auf die Wirkung der Handwerksnovelle zurückführen. Auch in den nicht von der Novelle betroffenen (unverändert zulassungspflichtigen) A-Handwerken zeigt sich mit dem Rückgang von 4.601 (2019) auf 4.260 (2020) eine negative Tendenz von 7 %. Der Rückgang geht speziell auf die gesunkene Gründungsdynamik zu Beginn der Corona-Krise (Kalenderwochen 13 bis 16) zurück. Anschließend zeigen sich aber Erholungseffekte und der Verlauf der Zeitreihen wird 2019 und 2020 nahezu identisch.

## Gründungsdynamik und Corona-Krise

Der Gesamteffekt der gesunkenen Gründungsdynamik 2020 ist somit nicht nur auf die Effekte der Handwerksnovelle zurückzuführen. Zwar sind die Rückgänge in den von der Novelle primär betroffenen Ausbauhandwerken am stärksten (vgl. Abbildung 3). Die Auswertung der Eintragungsstatistiken nach Konjunkturgruppen des Handwerks zeigt jedoch einen Rückgang der Gründungsdynamik

3 Die Eintragungszahlen umfassen neben Gründungen auch Ummeldungen (Rechtsform-, Tätigkeits- sowie Standortwechsel). Zur Vereinfachung werden hier die Begriffe Anmeldung, Eintragung und Gründung synonym verwendet.

Abbildung 2

**Gründungsdynamik nach Gruppen und Kalenderwochen (Januar bis August 2019, 2020)**

Index, 1. Januarwoche 2020 = 100

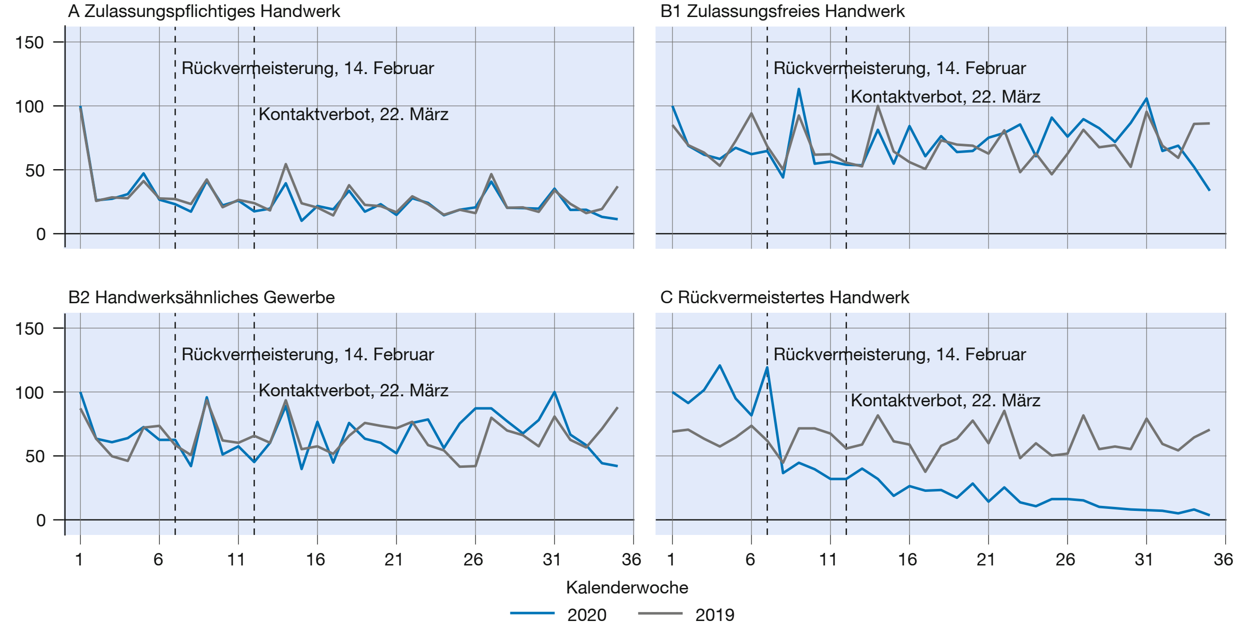

Quelle: Sonderabfrage bei 13 Handwerkskammern, August/September 2020.

auch in den von der Rückvermeisterung nicht betroffenen Gewerbegruppen. So verzeichnen das Lebensmittelgewerbe (-25 %), das Gesundheitsgewerbe (-13 %) und das Kfz-Gewerbe (-10 %) geringere Eintragungswerte als im Vorjahr.[4] Die gesunkene Gründungsdynamik wird damit in denjenigen Branchen des Handwerks sichtbar, für die auch die Handwerksberichterstattung des Statistischen Bundesamts die stärksten Umsatzrückgänge im 2. Quartal 2020 meldet. Nur die Handwerke für den privaten Bedarf zeigen distinktive Muster: Zwar waren sie von starken Umsatzrückgängen betroffen, die Gründungszahlen haben sich jedoch nach der Schwächephase zu Beginn der Krise schnell erholt, sodass der Gesamteffekt für den Zeitraum März bis August bei -3 % liegt. Womöglich haben in diesen Branchen die in der Krise entstandenen Nachfrageüberhänge (z.B. im Friseurhandwerk) die Gründungsabsichten positiv beeinflusst. Sehr robust zeigte sich schließlich in der Krise das Bauhauptgewerbe, bei dem keine systematischen Unterschiede zum Vorjahr feststellbar sind.

## Deutlicher Rückgang der Abmeldungen

Die Statistik der Abgänge (vgl. Abbildung 4) zeigt einen anderen Verlauf als die der Zugänge: Die meisten Abmeldungen erfolgen zum Jahresende bzw. im ersten Quartal des Jahres. Damit wäre mit deutlicheren Spuren der aktuellen Wirtschaftskrise auch erst zum Jahreswechsel zu rechnen. Allerdings lässt die Zeitreihe für 2020 (Januar bis August) erkennen, dass bereits ab April 2020 eine deutlich reduzierte Austragungsdynamik feststellbar ist, die bis August 2020 unverändert anhält. Zwischen April und August 2020 liegen die Austragungswerte durchgehend unter dem Niveau der Werte in den übrigen betrachteten Jahren. Damit kam es im Handwerk im Zuge der aktuellen Krise nicht zum Anstieg der Marktaustritte, sondern zu einer Reduktion. Das unerwartete Ergebnis der gesunkenen Marktaustritte im Abschwung lässt sich nur mit der Wirkung der wirtschaftspolitischen Stabilisierungsmaßnahmen im Zuge der Corona-Krise erklären. Offenbar konnten die Soforthilfeprogramme des Bundes und der Länder den Unternehmensbestand so weit stabilisieren, dass eine Insolvenzwelle trotz der Umsatzrückgänge und Liquiditätsprobleme bislang ausblieb und sogar mehr Unternehmen am Markt verblieben, als dies in den Vorjahren der Fall war.[5] Entscheidend für die endgültige Bestimmung der Auswirkungen der aktuellen Krise wird aber die Entwicklung um den Jahreswechsel bzw. mit dem Ende der Aussetzung der Insolvenzantragspflicht sein.

4 Die Unterschiede wurden für die Eintragungswerte von März bis August berechnet, also ab Beginn der Corona-Krise bis Ende des Erfassungszeitraums im Datensatz.

5 In der Gesamtwirtschaft zeigen sich ebenfalls gesunkene Gewerbeabmeldungen im Zuge der Corona-Krise (Günterberg, Kay und Kranzusch, 2020).

Abbildung 3

**Gründungsdynamik nach Konjunkturgruppen und Kalenderwochen (Januar bis August 2019, 2020)**

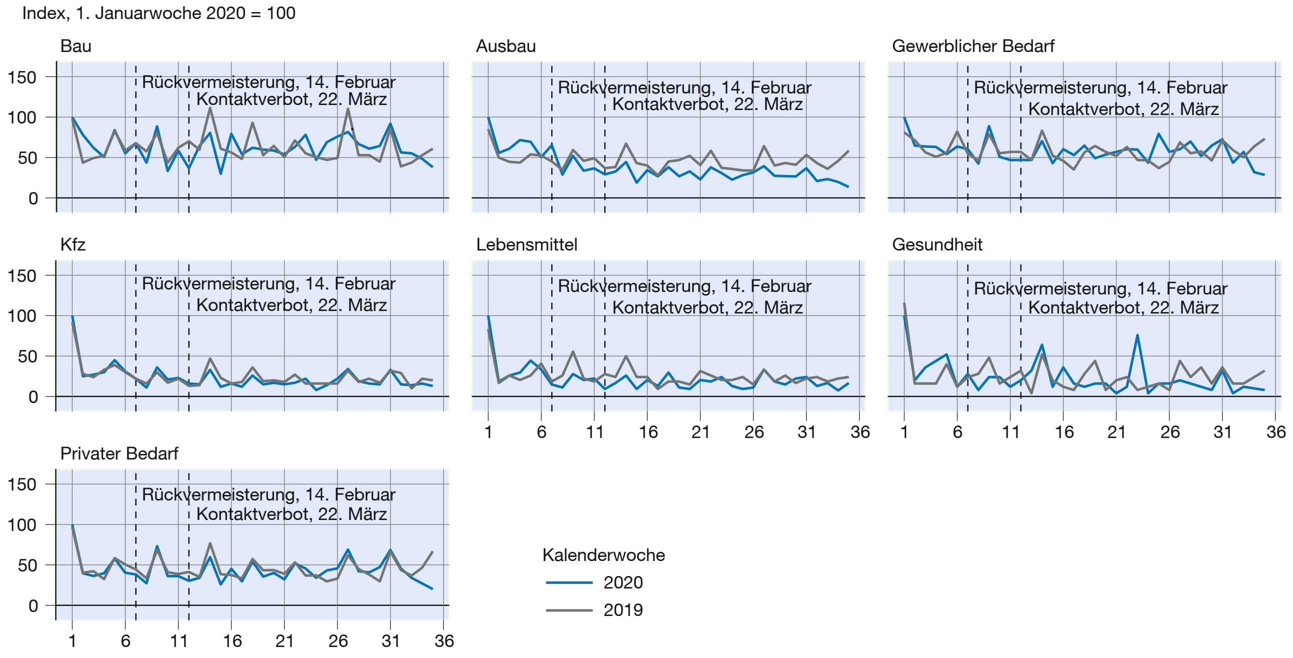

Quelle: Sonderabfrage bei 13 Handwerkskammern, August/September 2020.

Nimmt man die Unterscheidung nach den Anlagen der Handwerksordnung und Konjunkturgruppen des Handwerks vor, dann wird deutlich, dass die rückläufige Austragungsdynamik, anders als bei den Eintragungsdaten, unabhängig vom Zulassungsstatus und von der Konjunkturgruppe erkennbar ist. Während die Rückgänge bei den Eintragungsdaten in erster Linie bei den rückvermeisterten Handwerken und, in geringerem Umfang, bei den durchgehend zulassungspflichtigen Handwerken feststellbar waren, zeigen sich bei den Austragungen durchgehend rückläufige Entwicklungstendenzen.

## Fazit

Trotz erheblicher Umsatzrückgänge zeigte sich das Handwerk in der Corona-Krise ausgesprochen robust. Es kam zu keiner Entlassungswelle; die Betriebe ließen wie in der letzten Wirtschaftskrise 2008/2009 ein beharrendes Beschäftigungsverhalten erkennen und hielten an ihren Angestellten fest. Auch der Betriebsbestand konnte keinen massiven Verlust an Gründungen und keinen Anstieg der Betriebsaufgaben verzeichnen. Die Gründungstätigkeit ließ zwar zu Beginn der Krise nach, rasch zeigten sich jedoch Erholungseffekte. Zugleich kam es zu einem starken Rückgang der Marktaustritte, was die Betriebsbestände im Krisenverlauf zwar stabilisierte, jedoch Nachholeffekte mit dem Auslaufen der öffentlichen Hilfe- und Stabilisierungsprogramme erwarten lässt.

Auf Ebene der einzelnen Handwerke gestaltete sich die Situation sehr unterschiedlich. Als krisenresistent hat sich das Baugewerbe erwiesen. Gestützt wurde die positive Entwicklung im Baugewerbe in erster Linie durch den hohen Auftragsbestand vor Beginn der Pandemie. Dank der guten Witterungsverhältnisse und der vergleichsweise guten Umsetzbarkeit der Abstandsregeln auf Baustellen konnte der Baubetrieb auch im Lockdown erhalten bleiben. Handwerke für den privaten Bedarf (z. B. Friseurgewerbe) erlebten zwar tiefe Einschnitte zu Beginn der Krise, profitierten jedoch von den entstandenen Nachfrageüberhängen. Tiefere Spuren hat die Krise in erster Linie im Lebensmittelgewerbe, Gesundheitsgewerbe und dem Kfz-Gewerbe hinterlassen. In diesen Gewerbegruppen lassen sich sowohl starke Umsatzrückgänge als auch eine gesunkene Gründungsdynamik verzeichnen.

Die Gründungsdynamik im Ausbaugewerbe wurde schließlich in erster Linie durch die Auswirkungen der Handwerksnovelle 2020 geprägt. Die Eintragungsstatistiken der Kammern zeigen für die Gruppe der rückvermeisterten Handwerke eine Gründungswelle vor Inkrafttreten der Novelle und einen deutlichen Rückgang der Eintragungszahlen ab Mitte Februar 2020. Die Veränderungsraten in den Meldestatistiken sind insgesamt im Ausbaugewerbe am höchsten, was auf die Überlagerung der Effekte der Corona-Krise und der Effekte der Rückvermeisterung in den betroffenen Handwerken zurückzuführen ist.

Abbildung 4

**Austragungen nach Monat und Jahr, Januar 2007 bis August 2020**

Quelle: Sonderabfrage bei 13 Handwerkskammern, August/September 2020.

Bislang ist nicht davon auszugehen, dass die Corona-Krise eine nachhaltig verminderte Gründungstätigkeit und damit eine nachlassende Erneuerungsfähigkeit des Handwerks versursacht hat. Nach einer sichtbaren Erholungsphase im Sommer nehmen jedoch aktuell die Risiken und Unsicherheiten zu. Erschwerend wirkt nun, dass die Auftragsreichweiten im Handwerk im Zuge der Corona-Krise zurückgegangen sind und die innerbetrieblichen Reserven bereits in der ersten Lockdown-Phase stark beansprucht wurden. Unklar ist bislang auch, wie viele Betriebe mit dem Auslaufen der staatlichen Unterstützungsmaßnahmen den Markt verlassen werden. Es bleibt damit offen, ob die bisherige Resilienz des Handwerks auch im weiteren Krisenverlauf Bestand haben wird.
